# Exposure Assessment of Multiple Mycotoxins and Cumulative Health Risk Assessment: A Biomonitoring-Based Study in the Yangtze River Delta, China

**DOI:** 10.3390/toxins13020103

**Published:** 2021-02-01

**Authors:** Qingwen Huang, Keqiu Jiang, Zhanmin Tang, Kai Fan, Jiajia Meng, Dongxia Nie, Zhihui Zhao, Yongjiang Wu, Zheng Han

**Affiliations:** 1 College of Pharmaceutical Sciences, Zhejiang University, Hangzhou 310058, China; huangqingwen@zju.edu.cn (Q.H.); jiangkeqiu@zju.edu.cn (K.J.); 2 Institute for Agro-Food Standards and Testing Technology, Shanghai Key Laboratory of Protected Horticultural Technology, Laboratory of Quality and Safety Risk Assessment for Agro-Products (Shanghai), Ministry of Agriculture, Shanghai Academy of Agricultural Sciences, Shanghai 201403, China; kyatang@163.com (Z.T.); fankai@saas.sh.cn (K.F.); mengjiajia@saas.sh.cn (J.M.); niedongxia@saas.sh.cn (D.N.); zhaozhihui@saas.sh.cn (Z.Z.)

**Keywords:** mycotoxins, biomonitoring, cumulative risk assessment, hazard index, combined margin of exposure, urine

## Abstract

The extensive exposure to multiple mycotoxins has been demonstrated in many countries; however, realistic assessments of the risks related to cumulative exposure are limited. This biomonitoring study was conducted to investigate exposure to 23 mycotoxins/metabolites and their determinants in 227 adults (aged 20–88 years) in the Yangtze River Delta, China. Eight mycotoxins were detected in 110 urine samples, and multiple mycotoxins co-occurred in 51/227 (22.47%) of urine samples, with deoxynivalenol (DON), fumonisin B1 (FB1), and zearalenone (ZEN) being the most frequently occurring. For single mycotoxin risk assessment, FB1, ZEN, aflatoxin B1 (AFB1), and ochratoxin A (OTA) all showed potential adverse effects. However, for the 12 samples containing DON and ZEN, in which none had a hazard risk, the combination of both mycotoxins in two samples was considered to pose potential endocrine disrupting risks to humans by hazard index (HI) method. The combined margin of exposure (MOE_T_) for AFB1 and FB1 could constitute a potential health concern, and AFB1 was the main contributor. Our approach provides a blueprint for evaluating the cumulative risks related to different types of mycotoxins and opens a new horizon for the accurate interpretation of epidemiological health outcomes related to multi-mycotoxin exposure.

## 1. Introduction

Mycotoxins are toxic secondary metabolites produced by various toxic fungal species. More than 400 mycotoxins were identified, including aflatoxins (AFs), fumonisins (FUMs), ochratoxin A (OTA), zearalenone (ZEN), and trichothecenes (TCTs), which exhibited high toxicities and impacted human and animal health (e.g., mutagenic, teratogenic, and even carcinogenic activities) [1,2]. Therefore, the maximum acceptable levels [3] and the health-based guidance values (i.e., tolerable daily intakes (TDI)) have been set by the European Commission and the European Food Safety Authority (EFSA) [4,5,6,7,8].

Humans are exposed to multiple mycotoxins via the environment and daily consumer products. These mycotoxins are quickly metabolized as metabolites/prototypes and are excreted in urine [9]. Recently, urinary metabolites/prototypes have been successfully used as indicators of mycotoxins exposure in many human biomonitoring studies (HBM), which have been performed in Africa, Europe, Asia, and America [10,11,12,13]. In China, most previously conducted investigations were mainly directed toward a single class of mycotoxins, and only one study conducted in 2019 investigated multiple mycotoxins exposure in 260 rural residents (aged 18–66 years) in Nanjing [12]. More data covering larger representative areas are necessary to reveal the real health risks associated with mycotoxins in China.

Given the fact that consumers are exposed to multiple mycotoxins through various routes [14], the cumulative risks related to multiple mycotoxins have attracted more and more attention. Previous studies have proposed the idea that, even if two or more toxins are present in concentrations below those considered safe, exposure to the mixture may result in a cumulative toxic effect [15,16]. However, these bio-monitoring paradigms only considered the adverse effects of one mycotoxin and did not adequately address the overall mycotoxin exposure or assess the realistic risks of combined exposure to multiple mycotoxins. Recently, several models including hazard index (HI), combined margin of exposure (MOE_T_), and relative potency factors/toxic equivalency factors (RPF/TEF) have been established to assess the cumulative risk of co-occurring contaminants based on their chemical composition and knowledge of the toxicities [17,18,19]. Of these, the HI model is defined as the sum of the hazard quotient (HQ) for each individual compound in the mixture with no genotoxic or carcinogenic properties, while the margin of exposure (MOE) is proposed for the risk assessment of genotoxic and carcinogenic substances, and MOE_T_ is usually used for the cumulative risk assessment [20].

In the present study, we carried out a study to determine the levels of 23 mycotoxins/metabolites in first morning urine samples in a cohort of 227 adults living in the Jiangsu-Zhejiang-Shanghai area. The relationships between mycotoxins exposure and the dietary habits of residents as well as other elements, e.g., age, sex, and body mass index (BMI), were investigated to better understand the causal factors of the health risks to Yangtze River Delta populations. In order to evaluate the cumulative mycotoxins exposure, HQ, HI, MOE, and MOE_T_ models based on the targeted organs and the toxicity effect of typical mycotoxins were proposed to accurately interpret epidemiological health outcomes so as to set up effective prevention and control measures.

## 2. Results

### 2.1. Population Characteristics

**Figure 1:**
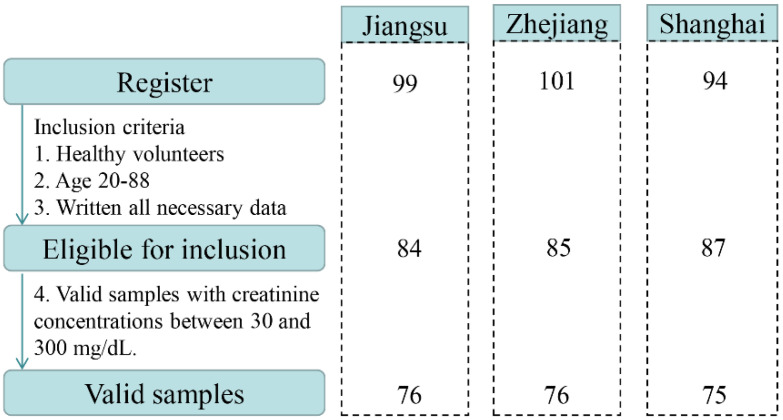
Flow chart describing the selection procedure of the studied samples.

A total of 294 volunteers, aged 7–88 years, were enrolled in the study. Based on the exclusion criteria, 38 recruited participants were excluded (age < 19 years, incomplete questionnaire, health problems). Overall, 256 healthy subjects returned urine samples and completed questionnaires between March and September 2019. After further validation, 29 urine samples were not eligible due to Cr concentrations <30 mg dL^−1^ or >300 mg dL^−1^. Finally, urine samples from 227 adults in the Yangtze River Delta, China were selected and used for the biomonitoring study. A flow chart describing the selection procedures is illustrated in Figure 1. The distributions of participants by sex, age, and BMI in the three sampling regions are presented in Table S1. Of 227 participants recruited for this study, 50.2% were male (n = 114) and 49.8% were female (n = 113). The mean age of all participants was 45.4 ± 17.5 years ranging from 20 to 88 years. The average BMI of the population was 23.1 ± 3.2 kg m^−2^.

### 2.2. Prevalence of Mycotoxins in Urine 

In total, 227 urine samples were analyzed for the presence of 23 mycotoxins/metabolites, and eight analytes were detected where FB1, ZEN, and DON and its metabolites were the most frequently measured in the urine samples. The prevalence and the levels of these mycotoxin biomarkers are illustrated in Table 1 (uncorrected and corrected by Cr). Only data above the limit of detection (LOD) were included. The quantification of DON-15-GlcA was based on the standard concentration curve of DON-3-GlcA as described in a previous study [21]. 

DON and its metabolites including DON-3-GlcA and DON-15-GlcA were the most prevalent (prevalence of 12.33%, 10.13%, and 30.40%, respectively), and their concentrations ranged from 0.50 to 35.00 ng mg^−1^. Their prevalence and concentrations were lower than those reported in populations in China (Henan, Sichuan), UK, Portugal, and Belgium [21,22,23,24]. The low levels of exposure to DON in this study may be due to the minimal occurrence and concentration of DON in cereal and oil products in the Yangtze Delta region [25].

Urinary AFM1 is well established as a biomarker of recent ingestion of AFB1 [26] and, in this study, the prevalence of AFM1 (2.20%) was lower than that of other mycotoxins, confirming critical and efficient control of AFB1 in the Yangtze River Delta. The mean urinary AFM1 concentration in our study was higher than the levels reported in Brazilian subjects (0.02 ng mg^−1^ Cr) [27] but similar to those reported in adults in Chinese studies (0.278 ng mg^−1^ Cr, 0.250 ng mg^−1^) [12] as well as in Nigeria (0.4 ng mg^−1^) [28] and Malaysia (0.64 ng mg^−1^) [29]. Considering that AFM1 is a specific biomarker of AFB1 exposure, is a class I carcinogen [30], and is harmful even at lower concentrations, the concentration of aflatoxins should be as low as reasonably achievable [31]. These results confirmed that it is necessary to continuously pay attention to the prevention and the control of AFB1 pollution in the Jiangsu-Zhejiang-Shanghai area.

With regard to OTA, its prevalence (3.52%) in the current study was markedly higher than that found in Nanjing, where OTA was detected in 1.2% of urine samples [12]. Differences in exposure may be attributed to different sample collection times and variable environmental conditions, e.g., high temperate and moist climate [27]. In European countries, such as Germany, Czech Republic, and Belgium, OTA was detected in urine samples at a prevalence ranging from 35% to 100% [11,21,32]. This higher prevalence could be explained by the different dietary habits in European countries, such as coffee and red wine consumption, favorable matrices for fungal growth and OTA production [33,34], which are different to those in China.

FB1, classified as a group 2B human carcinogen by International Agency for Research on Cancer (IARC) [30], was detected in 12.33% of urine samples with a mean value of 1.12 ng mL^−1^, which was higher than that detected in Portugal (3%, 0.24 ng mL^−1^) and Haiti (4%, 0.44 ng mL^−1^) [22,35] but significantly lower than those reported in children in Cameroon (11%, 2.96 ng mL^−1^) and Nigeria (13.3%, 4.6 ng mL^−1^) [26,28]. The higher prevalence and concentration in Africa and Asia could be explained by the different dietary habits as the populations in these regions consume a high percentage of their calories as corn meal, and this often coincides with the growing areas where mycotoxin contamination may be high [36]. In addition, children exhibited higher consumption relative to body weight when compared to adults, which is a cause for concern in Cameroon and Nigeria.

The results also showed that 11.89% of urine samples were contaminated by ZEN. ZEN-14-GlcA, the metabolite of ZEN, was detected in two urine samples in this study. The presence of ZEN and its metabolite was significantly lower than that found in previous biomonitoring studies in Bangladesh (ZEN 100%, 0.028 ng mL^−1^; α-ZEL 100%, 0.198 ng mL^−1^; β-ZEL 18%, 0.013 ng mL^−1^) [37], Germany (ZEN 100%, 0.10 ng mL^−1^; α-ZEL 100%, 0.16 ng mL^−1^; β-ZEL 18%, 0.05 ng mL^−1^) [38], and Nigeria (ZEN 81.7%, 0.75 ng mL^−1^; α-ZEL 4.2%, 1.27 ng mL^−1^; and β-ZEL 5.8%, 0.88 ng mL^−1^) [39]. These differences in prevalence rates, average concentration, and concentration range may be the result of the analytical methods employed as well as environmental and food storage conditions.

In addition, one or more analytes were detected in 110/227 (48.46%) urine samples (Table S2), verifying frequent exposures to mycotoxins. Detectable levels of two co-occurrent mycotoxins were found in 30 samples (13.22%). The most common combinations were DON+DON-15-GlcA, DON-3-GlcA+DON-15-GlcA, and DON-15-GlcA+ZEN, which were found in 12 (5.29%), 7 (3.08%), and 4 (1.76%) samples, respectively. Among the 13 samples (5.73%) with three mycotoxins, ZEN and DON and their metabolites were the most frequently detected. Four different mycotoxins were simultaneously found in eight urine samples, demonstrating that humans are commonly exposed to multiple mycotoxins.

### 2.3. Differences by Residential Area, BMI and Demographic Variables

The exposure levels to some mycotoxins differed due to residential area, sex, age, and BMI. These findings might be attributed, at least in part, to dietary characteristics, body fitness, and levels of mycotoxin contamination in food. The concentrations of mycotoxins in Jiangsu, Zhejiang, and Shanghai were slightly different (Table 1). OTA was less likely to be detected in Shanghai than in Jiangsu and Zhejiang. Jiangsu showed higher levels of urinary FB1, DON, and ZEN than the other two regions. The differences in the mean concentrations of DON and its metabolites in the three regions were significant (*p* < 0.05) when the urinary concentrations were corrected and uncorrected for Cr. This might be because Jiangsu province is an important wheat growing region located in China’s north-south transition zone and spans two major ecological regions suitable for wheat cultivation [40], which are easily infected by *Fusarium* species and are vulnerable to contamination by mycotoxins. In comparison, Zhejiang with fertile soils and abundant water resources is one of the world’s most productive rice growing regions [41], while in Shanghai, there is only a small amount of cultivated land, which is also mainly used for rice cultivation, certainly resulting in a lower level of contamination by *Fusarium* mycotoxins. There are no obvious differences in the mean concentrations of other mycotoxins in the three regions (*p* > 0.05). In addition, the higher prevalence and concentration of mycotoxins in the current study compared to those reported by Fan et al. [12] may be due to the different environmental conditions and climates between March–September 2019 and January–March 2017.

**Figure 2:**
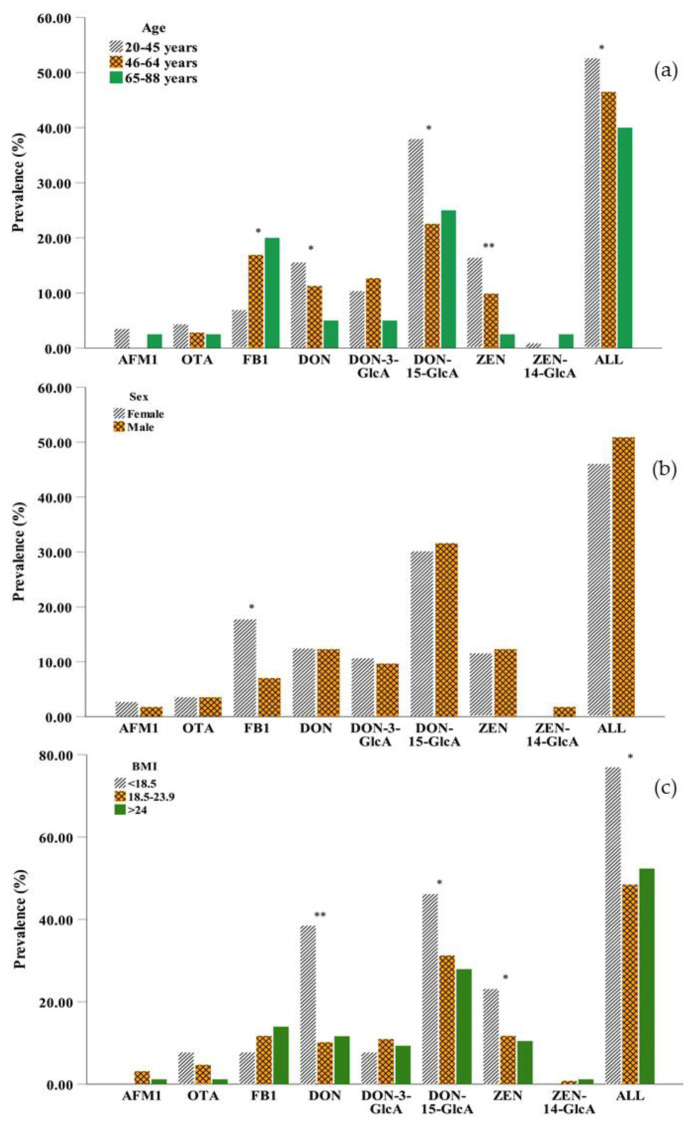
Comparison of the prevalence of eight mycotoxins in urine (**a**) among different age groups: 20–45 years (*n* = 116), 46–64 years (*n* = 71), 65–88 years (*n* = 40); (**b**) between males (*n* = 114) and females (*n* = 113) in the three regions and (**c**) in the different BMI groups: <18.5 kg m^−2^ (*n* = 13), 18.5–23.9 kg m^−2^ (*n* = 128), >24 kg m^−2^ (*n* = 86). (AFM1: aflatoxin M1; OTA: ochratoxin A; FB1: fumonisin B1; DON: deoxynivalenol; DON-3-GlcA: deoxynivalenol-3-glucuronide; DON-15-GlcA: deoxynivalenol-15-glucuronide; ZEN: zearalenol; ZEN-14-GlcA: zearalenone-14-glucuronide; ALL: mycotoxin contamination in any case; *: 0.01 < *p*-value < 0.05; ** *p*-value < 0.01 by the chi-square test).

With regard to age, generally, DON and ZEN were more likely to be detected in young adults (20–45 years) than in the other age groups, and their prevalence decreased with increasing age (Figure 2a). The two main metabolites of DON, DON-3-GlcA, and DON-15-GlcA, were less likely to be detected in older persons (65–88 years) and middle aged adults (46–64 years), respectively, while the metabolite of ZEN, ZEN-14-GlcA was detected in trace amounts in all samples and was not found in middle-aged adults. In general, mycotoxins were more frequently detected in young persons than in middle-aged adults or older persons with significant differences observed (*p* < 0.05), because younger adults have higher food consumption compared to older adults [42]. However, the differences in the concentrations of mycotoxins were not significant between the different age groups (Table S3). Different from other mycotoxins, the lowest prevalence and concentration of FB1 in urine were observed in 20–45 years group due to the rapid elimination of this toxin resulting in a low bioavailability [43].

The effect of sex was necessarily considered [44], and slight differences in mycotoxin exposure were observed between men and women (Figure 2b and Table S3). Compared to males, females had a higher prevalence of AFM1 and FB1 but a lower prevalence ZEN. The differences were not significant expect FB1 (*p* > 0.05). The prevalence of OTA, DON, DON-15-GlcA, and ZEN was more likely to be detected in the underweight group (BMI < 18.5 kg m^−2^) than in the normal weight group (BMI 18.5–23.9 kg m^−2^) and the overweight group (BMI > 24 kg m^−2^). FB1 was more likely to be detected in the overweight group than in the other two groups (Figure 2c), which might be due to higher food consumption. However, the underweight group (BMI < 18.5 kg m^−2^) in our study preferred to drink beverages such as coffee and tea, which are sensitive to OTA contamination. In addition, due to the short half-lives of mycotoxins (DON and ZEN and its metabolites), they were less likely to be found in overweight adults, which could be caused by the relatively higher basal metabolic rate [45].

### 2.4. Correlation between Mycotoxins and Food Consumption

To identify the possible sources and the determinants of mycotoxins exposure, the correlations between urinary concentrations and food consumption were analyzed based on the information in the food questionnaires. As shown in Table 2, urinary AFM1 concentrations showed a high correlation (*r*_s_ = 0.866) with the consumption of nuts and seeds, as these foods are frequently contaminated by aflatoxins [46]. Urinary OTA concentrations also showed a high positive correlation (*r*_s_ = 0.778, *p* < 0.05) with the consumption of beverages such as coffee and tea, which are easily and frequently contaminated by OTA [33,34]. Only a few moderate correlations were observed between AFM1 levels and the consumption of milk and dairy products (*r*_s_ = 0.289), wheat (*r*_s_ = 0.224), and beverages such as coffee and tea (*r*_s_ = −0.224), which was consistent with previous studies [47]. Milk and dairy consumption was negatively correlated with the OTA levels (*r*_s_ = −0.275) but positively correlated with FB1 levels (*r*_s_ = 0.362). There were no statistically significant correlations (*p* < 0.05) between mycotoxins exposure and food consumption except OTA and beverages such as coffee and tea, which was consistent with several previous studies [48,49] and might be caused by the heterogeneous distributions of mycotoxins in foodstuffs, various routes of mycotoxin exposure, and the inaccurate dietary intake in questionnaires [50].

### 2.5. Risk Assessment of Single Mycotoxins 

The dietary intake of AFB1, FB1, OTA, DON, and ZEN was estimated using the concentrations of biomarkers measured in urine samples (Table 3). For AFB1, a mean probable daily intake (PDI) of 0.62 µg/kg·bw/day and a maximum PDI of 1.03 µg/kg·bw/day were determined when the excretion rate of 1.5% was considered [51], which were higher than those in Brazil (mean intake = 0.001 µg/kg·bw/day) and in Haiti (mean intake = 0.03 µg/kg·bw/day) [27,35] but similar to Nigeria (mean intake = 0.67 µg/kg·bw/day) [28], which posed a potential public concern for residents in the Jiangsu-Zhejiang-Shanghai area. For OTA, the mean PDI value was 0.14 µg/kg·bw/day, which was higher than the report on a Portuguese population [22]. However, Franco et al. [27] reported a greater prevalence of OTA and a lower mean PDI value (0.031 µg/kg·bw/day) due to a high excretion rate (50%). The MOE values of AFB1 and OTA were lower than 200 in a high risk-range, leading to high concern for these mycotoxins.

In adults with positive detection of mycotoxins, 18.93% showed potential health risks with HQ values > 1. FB1 exhibited the highest hazard effect, and 12.33% of participants were exposed to this mycotoxin with HQ values > 1 and emerged with higher adverse effects than in Italy, Portugal, and Brazil [22,27,35]. This could be associated with the high occurrence of FB1 (57%) in maize-based products in China [52]. However, in this study, none of the participants showed a hazard risk in relation to DON, and the mean PDI was 0.08 µg/kg·bw/day, which was considered to be as safe as that in the overall population studied. In addition, higher HQ levels (> 1) for ZEN (0.88% of participants) were less common in adults in the current study than in the Portuguese (24%) [22] and the German (1.67%) [38] populations, which might be due to the various dietary habits as well as toxicokinetics and toxicodynamics of the populations from different countries.

### 2.6. Cumulative Risk Assessment of Co-Occurring Mycotoxins

As previously referenced, humans are simultaneously exposed to multiple mycotoxins, and our results on the occurrence of various mycotoxins in urine samples confirmed this. However, until now, the risk assessment of mycotoxins was normally performed on each single substance, leading to an underestimation of risk. To accurately evaluate the cumulative risks of multiple mycotoxins, HI and MOE_T_ models were proposed in this study, which are primary and frequently used regulatory approaches for the risk assessments of mixtures of toxicologically similar chemicals [20]. Considering the number of mycotoxins present in urine samples and all possible mixtures, the co-occurring mycotoxins were grouped with respect to their similar toxicological effects on a target organ (or system). To accommodate the consistency required for the risk assessment of mixtures, the reference values for mixed mycotoxins should be in the orientation to the same toxicity endpoints, which might be different to the values used for the individual risk assessment [53]. The toxicological data and the key studies/critical effects were obtained from EFSA [4,5,6,7,8] and are shown in Table S4. AFB1 and FB1 displayed qualitatively similar toxicological profiles with hepatotoxicity being the common toxic effect, which could lead to hepatocellular carcinomas or an increased incidence of megalocytic hepatocytes at low dose. With regard to the endocrine activities of DON and ZEN, ZEN had the strongest estrogenic potency, and DON showed estrogenic activity in vitro [54]. DON and ZEN could promote germ cell degeneration, sperm retention, and abnormal nuclear morphology or disturbance of the estrous cycle, ovulation, conception, and implantation in vivo. Cumulative risk assessments were subsequently performed for hepatotoxicity (AFB1 and FB1) and endocrine activity effects (DON and ZEN), respectively.

Two urine samples co-contaminated with AFB1 and FB1 (Group 1) and twelve samples containing both DON and ZEN (Group 2) were selected for cumulative health risk assessment based on the MOE_T_ and the HI models, respectively (Table 4). Due to the risk decreasing as the MOE increased [55], in two urine samples with co-occurrence of AFB1 and FB1, AFB1, which is considered the most potent aflatoxin, revealed a MOE below 10,000 in a high risk-range, and FB1 affecting the same target organ would further strengthen the harm. A number of articles also have reported the combined effects of AFB1 and FB1 in experimental animals [56,57]. Although, in the present experiment, there are few samples which were co-contaminated by AFB1 and FB1, their co-existence and combined toxicity still need to be closely monitored due to the fact that they are two of the toxicologically most relevant mycotoxins occurring in maize [56]. Due to the co-occurrence of DON and ZEN in different foodstuffs [25,58], these two mycotoxins were also the most frequently co-occurring mycotoxins in urine samples. When only one mycotoxin was considered, all 12 samples were found to be safe (HQ < 1), but when the combination of both were considered, two samples showed potential endocrine activity risks to humans. Due to the high prevalence and the low TDI value, ZEN was obviously the larger contributor to the total risk compared to DON. The above observations clearly indicated that risk assessments of single mycotoxins were strikingly insufficient and underestimated numerous adverse effects; therefore, the cumulative risks of different mycotoxins should be an important research core, which would attract more attention and further studies.

The main limitations of this study also needed to be underlined. First, dietary intake, weight, socio-demographic, and lifestyle information were self-reported; therefore, misreporting could not be excluded and may be related to other factors, including socioeconomic status, occupation, perceived weight status, and level of physical activity. Second, due to the short half-lives of some mycotoxins (such as DON, DON-3-GlcA, and α-ZEL) [22,59] included in this study, one spot urine is unlikely to monitor every exposure to mycotoxins, which could also lead to an underestimation of mycotoxins exposure. Thirdly, the pending elucidation of the mode of actions for the genotoxicity/carcinogenicity of OTA and FB1, an MOE of 10,000 to the neoplastic reference point, is warranted for the risk characterization of OTA and calculating the MOE_T_ with AFB1 and FB1. The assessment is more likely to overestimate than to underestimate the risk [8]. Further investigations are necessary to confirm the findings of this study.

## 3. Conclusions

This biomonitoring-based study was performed to assess the cumulative health risks of multiple mycotoxins in the Yangtze River Delta, China. The ubiquitous exposure of a broad segment of the investigated population to mycotoxins was observed, and 22.47% of adults were found to be co-exposed to two or more contaminants, mainly ZEN, DON, and their metabolites. The heterogeneity of mycotoxins exposure was identified, and several socio-demographic lifestyle characteristics, such as place of residence, age, sex, and BMI, were associated with mycotoxin body burden. Considering the high prevalence of co-contamination with multiple mycotoxins, MOE or HQ and HI or MOE_T_ models were put forward for risk assessment of exposure to single and multiple mycotoxins. Potential hazard effects were shown to be related to single mycotoxins, and furthermore, the integration of two mycotoxins clearly contributed to a greater health concern, which endowed the risks related to the mycotoxins within the TDI limits. For the 12 samples containing DON and ZEN, in which none had a hazard risk, the combination of both mycotoxins in two samples was considered to pose potential endocrine disrupting risks to humans. This is the first time an efficient strategy has been proposed for the quantitative assessment of cumulative health risks of multiple mycotoxins exposure using urinary biomarkers. This strategy successfully avoided underestimation of the health risks when exposed to multiple mycotoxins. Thus, an overhaul of legislation and control programs is required, as not only the risks of individual mycotoxins should be considered but also their synergic effects.

## 4. Materials and Methods

### 4.1. Reagents and Chemicals

Methanol and acetonitrile, both HPLC grade, were obtained from Merck (Darmstadt, Germany). Formic acid and ammonium acetate were obtained from Sigma-Aldrich (St. Louis, MO, USA). Water was purified using a Milli-Q Plus apparatus (18.2 Ω, Millipore, Billerica, MA, USA) prior to use. The standards of aflatoxin B1 (AFB1), aflatoxin B2 (AFB2), aflatoxin G1 (AFG1), aflatoxin G2 (AFG2), aflatoxin M1 (AFM1), aflatoxin M2 (AFM2), OTA, ochratoxin α (OTα), fumonisin B1 (FB1), T-2 toxin (T-2), HT-2 toxin (HT-2), deoxynivalenol (DON), 3-acetyldeoxynivalenol (3-ADON), 15-acetyldeoxynivalenol (15-ADON), fusarenon X (FUS-X), ZEN, α-zearalenol (α-ZEL), β-zearalenol (β-ZEL), α-zearalanol (α-ZAL), and β-zearalanol (β-ZAL) were purchased from Romer Labs (Union, MO, USA). The mycotoxin conjugates deoxynivalenol-3-glucuronide (DON-3-GlcA) and zearalenone-14-glucuronide (ZEN-14-GlcA) were prepared in our laboratory as described in a previous study [12]. The quantification of DON-15-GlcA was based on the standard concentration curve of DON-3-GlcA as described in a previous study [21].

### 4.2. Study Population 

Morning urine samples were obtained from 227 adults residing in the Yangtze River Delta, China between March 2019 and September 2019. Attempts were made to achieve a representative distribution for sex (male and female), age (20–45, 46–64, 65–88-years-old) [60], and region (Jiangsu, Zhejiang, and Shanghai). The participants were asked to complete a detailed questionnaire, which included demographics, health, and lifestyle factors. A food questionnaire on dietary intake the previous day was administered, in which typical foods such as wheat, maize, rice, vegetables and fruit, meat, nuts and seeds, milk and dairy products, and drink (including beverages such as coffee and tea) consumed by Jiangsu, Zhejiang, and Shanghai residents were clearly listed. The results for daily food intake in the participants are shown in Supplementary Table S5.

### 4.3. Urine Sample Preparation

The morning urine samples (~20 mL) were collected from the participants using 50 mL sterile centrifuge tubes (Corning, NY, USA). All urine samples were transported to the laboratory in chilled conditions after collection and stored in a freezer (−20 °C) within 6 h.

### 4.4. Biomarker Analysis

The extraction and the purification of 23 mycotoxins/metabolites in urine samples were performed as described in our previous study [12]. Briefly, each urine sample (200 μL) was measured accurately into a 1.5 mL centrifuge tube, and 1.0 mL of acetonitrile containing 1% formic acid was added. The sample was vortexed for 2 min and centrifuged at 12,000× *g* for 10 min at 4 °C. The acquired supernatant was dried by nitrogen stream, and then the residues were re-dissolved in 200 μL of acetonitrile/water containing 5 mmol L^−1^ ammonium acetate (20/80, v/v) and passed through a 0.22 μm syringe filters before ultra-high-performance-liquid chromatography tandem mass spectrometry (UHPLC-MS/MS) analysis. The instrumental conditions are detailed in Supplementary S1, and observed mass transitions and target-specific parameters are listed in Table S6.

To account for the variable dilutions of urine, urinary mycotoxin concentration was also reported as the adjusted concentration on the basis of creatinine (ng mg^−1^ creatinine (Cr)), the concentration of which was measured using an enzymatic reaction on a Roche Hitachi 912 Chemistry Analyzer (Roche Hitachi, Basel, Switzerland). According to the proposal of the World Health Organization (WHO) [61], samples too diluted (Cr. concentration < 30 mg dL^−1^) or too concentrated (Cr. concentration > 300 mg dL^−1^) were excluded. 

### 4.5. Estimated Dietary Exposure of Mycotoxins 

Probable daily intake (PDI) of mycotoxin was calculated according to Equation (1) [62]:

(1) $$\mathrm{PDI}\;(µg/\mathrm{kg}\cdot \mathrm{bw}/\mathrm{day})\;=\;\frac{C\times V\times 100}{W\times E}$$

where C is urinary biomarker concentration (μg L^−1^) (for DON, C_(DON)_ = C _(free DON)_ + (C _(DON-3-GlcA)_ + C _(DON-15-GlcA)_)*0.63), V is the mean volume of daily urine production (1.5 L day^−1^), W is body weight (kg), and E is mean urinary excretion rate (in percent) (1.5 for AFM1 [50], 2.5 for OTA [63], 0.5 for FB1 [64], 9.4 for ZEN [14], and 72 for DON [65]).

### 4.6. Risk Characterization 

#### 4.6.1. Risk Assessment of Single Mycotoxins

Two different approaches were performed for the risk assessment of individual mycotoxins based on their carcinogenicity. The MOE was applied for aflatoxins and OTA and was calculated as a ratio of benchmark dose lower confidence limit (BMDL_10_) and their exposure (as Equation (2)). Considering the carcinogenicity of OTA and AFB1, no level of exposure is considered safe, and the MOE was just calculated to help prioritize risk management actions. BMDL_10_ value were 0.4 µg/kg·bw/day for aflatoxins and 14.5 µg/kg·bw/day for OTA with neoplastic effects [7,8]. The magnitude of the MOE gives an indication of the risk level, and the Scientific Committee of EFSA and WHO have concluded that a MOE of 10,000 or more is of low concern for public health [20].

MOE = BMDL_10_/PDI (2)

For the remaining mycotoxins, HQ, used for assessing the adverse effects related to single mycotoxins, is the ratio of the PDI to the reference dose for relevant health endpoints [66], which could be the TDI (Equation (3)) or the value of the no observed adverse effect level (NOAEL) (Equation (4)). The uncertainty factor (UF) was set as 100 (10 × 10), which was interpreted as reflecting extrapolation from experimental animals to human (factor 10 for inter-species variability between man and animal) and extrapolation from an average human NOAEL to a sensitive human NOAEL (factor 10 for human or intra-species variability) [67]. When the HQ was <1, the exposure was considered to be within safe limits [20].

HQ_TDI_ = PDI/TDI (3)

HQ_NOVEL_ = PDI/NOAEL × UF (4)

#### 4.6.2. Cumulative Risk Assessment of Co-Occurring Mycotoxins 

Considering the co-occurrence of different mycotoxins, two groups were established with regard to toxicological similarity: Group 1, AFB1 and FB1 based on hepatotoxicity; Group 2, DON and ZEN based on endocrine activity (testicular toxicity and strong estrogenic potency). The MOE_T_ was utilized for Group 1 and the HI method for Group 2. The MOE_T_ was calculated as the reciprocal of the sum of the reciprocals of the individual MOEs (Equation (5)), and it was considered to be high concern for public health if MOE_T_ < 100. HI was the sum of the respective HQs for the individual mixture components of the same toxicity (Equation (6)). Significant adverse effects were speculated if HI > 1. The values of BMDL_10_, NOAELs, and TDIs used in the current study are presented in Table S4.

MOE_T_ = 1/(1/MOE_AFB1_ + 1/MOE_FB1_) (5)

HI = HQ_TDI,ZEN_ + HQ_NOVEL,DON_ (6)

### 4.7. Statistical Analysis 

All statistical analyses were performed using IBM SPSS Statistics software version 25 (SPSS Inc., Chicago, IL, USA) and Origin version 8.0 (Microcal, Inc., Northampton, MA, USA). For data analysis, urinary mycotoxin concentrations below the limit of detection (LOD) were replaced by zero, and the mycotoxins concentration values below the limit of quantification (LOQ) but above the LOD were replaced by 1/2 LOQ. As the data were not normally distributed, Fisher’s exact test and rank sum test were used to investigate the distributions of urinary mycotoxins by region, sex, age, and BMI. Spearman’s rank correlation coefficient (*r*_s_, ≤ 0.2 = poor; 0.2 ≤ 0.5 = fair; 0.5 ≤ 0.7 = moderate; 0.7 ≤ 0.9 = very strong) [22] was used for analysis of the relationships between two subgroups. When the *p*-value was <0.05, the differences were considered statistically significant.

## Figures and Tables

**Table 1:** Occurrence of mycotoxins/metabolites in human urine samples in the Yangtze River Delta, China.

Mycotoxins	All (*n* = 227)	Jiangsu (*n* = 76)	Zhejiang (*n* = 76)	Shanghai (*n* = 75)	*p*-Value ^1^
AFM1					
*n* (prevalence, %)	5 (2.20)	1 (1.32)	1 (1.32)	3 (4.00)	
Mean ± SD ^2^ (ng mL^−1^)	0.35 ± 0.17	0.10	0.43	0.41 ± 0.12	0.344
Range (ng mL^−1^)	0.10–0.55	0.10	0.43	0.31–0.55	
Mean ± SD (ng mg^−1^ Cr) ^3^	0.23 ± 0.07	0.12	0.27	0.26 ± 0.04	0.344
Range (ng mg^−1^ Cr)	0.12–0.29	0.12	0.27	0.21–0.29	
OTA					
*n* (prevalence, %)	8 (3.52)	2 (2.63)	1 (1.32)	5 (6.67)	
Mean ± SD (ng mL^−1^)	0.14 ± 0.06	0.16 ± 0.08	0.16	0.13 ± 0.06	0.717
Range (ng mL^−1^)	0.05–0.22	0.11–0.22	0.16	0.05–0.21	
Mean ± SD (ng mg^−1^ Cr)	0.21 ± 0.12	0.14 ± 0.11	0.28	0.22 ± 0.12	0.699
Range (ng mg^−1^ Cr)	0.05–0.37	0.06–0.22	0.28	0.05–0.37	
FB1					
*n* (prevalence, %)	28 (12.33)	15 (19.74)	10 (13.16)	3 (4.00)	
Mean ± SD (ng mL^−1^)	1.12 ± 0.40	1.18 ± 0.36	1.01 ± 0.35	1.17 ± 0.68	0.416
Range (ng mL^−1^)	0.51–1.96	0.51–1.76	0.51–1.68	0.75–1.96	
Mean ± SD (ng mg^−1^ Cr)	1.12 ± 0.74	0.88 ± 0.44	1.33 ± 0.98	1.56 ± 0.87	0.326
Range (ng mg^−1^ Cr)	0.30–3.34	0.30–1.82	0.37–3.34	0.57–2.20	
DON					
*n* (prevalence, %)	28 (12.33)	10 (13.16)	6 (7.89)	12 (16.00)	
Mean ± SD (ng mL^−1^)	1.79 ± 1.86	3.29 ± 2.39	1.63 ± 0.75	0.64 ± 0.34	0.000 *
Range (ng mL^−1^)	0.50–8.61	0.50–8.61	1.09–3.00	0.50–1.56	
Mean ± SD (ng mg^−1^ Cr)	1.50 ± 2.19	3.17 ± 3.05	0.95 ± 0.34	0.38 ± 0.15	0.000 *
Range (ng mg^−1^ Cr)	0.17–9.88	0.52–9.88	0.56–1.46	0.17–0.68	
DON-3-GlcA					
*n* (prevalence, %)	23 (10.13)	10 (13.16)	7 (9.21)	6 (8.00)	0.014 *
Mean ± SD (ng mL^−1^)	1.15 ± 1.00	1.79 ± 1.23	0.60 ± 0.26	0.73 ± 0.35	
Range (ng mL^−1^)	0.50–4.20	0.50–4.20	0.50–1.18	0.50–1.23	
Mean ± SD (ng mg^−1^ Cr)	1.10 ± 1.43	1.89 ± 1.90	0.45 ± 0.37	0.56 ± 0.31	0.004*
Range (ng mg^−1^ Cr)	0.20–6.99	0.53–6.99	0.20–1.22	0.32–1.18	
DON-15-GlcA					
*n* (prevalence, %)	69 (30.40)	22 (28.95)	19 (25.00)	28 (37.33)	
Mean ± SD (ng mL^−1^)	2.55 ± 4.68	5.09 ± 7.62	1.52 ± 1.47	1.26 ± 1.00	0.016*
Range (ng mL^−1^)	0.50–35.00	0.50–35.00	0.50–6.50	0.50–3.39	
Mean ± SD (ng mg^−1^ Cr)	2.16 ± 3.50	4.43 ± 5.45	1.19 ± 1.11	1.04 ± 0.82	0.033*
Range (ng mg^−1^ Cr)	0.17–20.77	0.30–20.77	0.22–3.79	0.17–3.53	
ZEN					
*n* (prevalence, %)	27 (11.89)	19 (25.00)	4 (5.26)	4 (5.33)	
Mean ± SD (ng mL^−1^)	0.97 ± 3.49	1.22 ± 4.16	0.40 ± 0.44	0.38 ± 0.56	0.868
Range (ng mL^−1^)	0.10–18.35	0.10–18.35	0.10–1.04	0.10–1.23	
Mean ± SD (ng mg^−1^ Cr)	0.65 ± 2.07	0.81 ± 2.46	0.22 ± 0.20	0.31 ± 0.27	0.771
Range (ng mg^−1^ Cr)	0.03–10.93	0.04–10.93	0.03–0.51	0.04–0.68	
ZEN-14-GlcA					
*n* (prevalence, %)	2 (0.88)	-	1 (1.32)	1 (1.33)	
Mean ± SD (ng mL^−1^)	0.69 ± 0.62	-	1.13	0.25	0.317
Range (ng mL^−1^)	0.25–1.13	-	1.13	0.25	
Mean ± SD (ng mg^−1^ Cr)	0.37 ± 0.18	-	0.50	0.24	0.317
Range (ng mg^−1^ Cr)	0.24–0.50	-	0.50	0.24	

^1^*p*-values obtained using the Kruskal–Wallis test, which compared mycotoxin concentrations in the three regions, * *p*-value < 0.05. ^2^ Only positive samples (≥limit of detection (LOD)) were considered for calculation of mean values. (LOD: aflatoxin M1 (AFM1), 0.05 ng mL^−1^; ochratoxin A (OTA), 0.05 ng mL^−1^; fumonisin B1 (FB1), 0.1 ng mL^−1^; deoxynivalenol (DON), 0.5 ng mL^−1^; DON-3-GlcA, 0.5 ng mL^−1^; zearalenone (ZEN), 0.1 ng mL^−1^; ZEN-14-GlcA, 0.2 ng mL^−1^). ^3^ Creatinine-adjusted concentration (ng mg^−1^ Cr).

**Table 2:** Correlations (*r_s_* and *p*-values) between urinary mycotoxin concentrations and food consumption.

Foods	AFM1		OTA		FB1		DON ^1^		ZEN	
	*r_s_*	*p*	*r_s_*	*p*	*r_s_*	*p*	*r_s_*	*p*	*r_s_*	*p*
Wheat	0.224	0.718	0.309	0.457	−0.355	0.064	−0.125	0.292	0.095	0.638
Maize	-	-	−0.247	0.555	0.192	0.328	0.057	0.631	−0.119	0.554
Rice	−0.053	0.933	−0.126	0.766	−0.111	0.573	−0.074	0.536	0.290	0.142
Vegetables and fruit	−0.154	0.805	−0.247	0.555	−0.191	0.331	−0.106	0.374	−0.089	0.660
Meat	0.000	1.000	−0.179	0.672	0.191	0.332	0.067	0.574	0.151	0.451
Nuts and seeds	0.866	0.058	0.109	0.797	−0.198	0.312	−0.178	0.132	0.127	0.529
Milk and dairy produce	0.289	0.638	−0.275	0.510	0.362	0.058	−0.067	0.576	−0.206	0.304
Beverages, coffee and tea	−0.224	0.718	0.778	0.023 *^2^	0.003	0.990	0.186	0.116	0.153	0.447

^1^ DON: the sum concentration of DON, DON-3-GlcA, and DON-15-GlcA. *^2^ *p* < 0.05.

**Table 3:** Risk assessments of individual mycotoxins by the margin of exposure (MOE) and the hazard quotient (HQ) in adults from the Yangtze River Delta, China (*n* = 227).

	Biomarker	Model	Mean PDI ^1^	N (%) ^2^	Median	P95	Max
AFB1	AFM1	MOE	0.62	2.20	0.67	2.14	2.50
OTA	OTA	MOE	0.14	3.52	110.31	217.05	270.67
FB1	FB1	HQ	5.51	12.33	5.24	8.58	10.5
DON	DON+DON-3-GlcA+DON-15-GlcA	HQ	0.08	0	0.03	0.28	0.85
ZEN	ZEN	HQ	0.20	0.88	0.13	1.18	13.78

^1^ Only positive samples (≥ LOD) were considered for the calculation of dietary intake. ^2^ N (%), percentage of samples posing health risks (percent in all, %).

**Table 4:** Cumulative risk assessment of co-occurring mycotoxins in adults using combined margin of exposure (MOE_T_) and hazard index (HI) models, respectively.

Group	Mycotoxin	Toxicological Effect	MOE/HQ		
			Mean	Max	N(n) ^1^
1	AFB1	hepatotoxicity	0.61	0.67	2 (2)
	FB1	hepatotoxicity	19.46	20.98	-
	MOE_T_^2^	hepatotoxicity	0.59	0.65	2 (2)
2	DON	male reproductive	0.02	0.04	0 (12)
	ZEN	estrogenic	0.37	0.99	0 (12)
	HI ^3^	endocrine effect	0.39	1.02	2 (12)

^1^ N(n): Number of HQ/HI values > 1 or MOE < 10,000 (total number of co-contaminated samples). ^2^ MOE_T_ = 1/(1/MOE_AFB1_ + 1/MOE_FB1_). ^3^ HI_endocrine effect_ = HQ_NOAEL, DON_ + HQ_TDI, ZEN_.

## Supplementary Materials

The following are available online at https://www.mdpi.com/2072-6651/13/2/103/s1, S1: Detailed UHPLC-MS/MS method, Table S1: The demographic characteristics of participants in the three sampling areas, Table S2: Co-occurrence of investigated mycotoxins and their metabolites in urine samples from Jiangsu, Zhejiang, and Shanghai., Table S3: *P*-value of mycotoxin concentration distribution by age, sex, and BMI using the rank sum test (*n* = 227),Table S4: Toxicities of FB1, OTA, DON, and ZEN from EFSA data, Table S5: Food consumption in the total population stratified by location, Table S6: LC-MS/MS parameters for the detection of targeted mycotoxins.
