# Supplementary material for: Exposure Assessment of Multiple Mycotoxins and Cumulative Health Risk Assessment: A Biomonitoring-Based Study in the Yangtze River Delta, China
Source: Toxins (Basel). 2021 Feb 1;13(2):103. doi: 10.3390/toxins13020103 (PMC7912756; doi:10.3390/toxins13020103)
Supplement: Supplementary file 1 [file toxins-13-00103-s001.pdf]

Supplementary Materials: Exposure Assessment of Multiple Mycotoxins and Cumulative Health Risk Assessment: A Biomonitoring-Based Study in the Yangtze River Delta, China

Qingwen Huang, Keqiu Jiang, Zhanmin Tang, Kai Fan, Jiajia Meng, Dongxia Nie, Zhihui Zhao, Yongjiang Wu and Zheng Han

S1. Detailed UHPLC-MS/MS Method

The method for accurate determination of 23 mycotoxins/metabolites in urine samples was performed via a Waters ACQUITY UHPLC system (Milford, MA, USA) coupled with an AB SCIEX QTRA® 5500 tandem mass spectrometer (AB SCIEX instruments, Foster City, Canada). Chromatographic separation was achieved on an Agilent Poroshell 120 EC-C_{18} column (3.0 mm × 100 mm, 2.7 μm) (Agilent, USA) with the mobile phase consisting of methanol (A) and 5 mmol/L ammonium acetate (B). A linear gradient elution program was designed as follows: initial 10% (A), 1 min 10% (A), 6 min 90% (A), 6.5 min 90% (A), 6.7 min 10% (A), and 8 min 10% (A) and held for a further 2 min for re-equilibration, yielding a total run time of 10 min. The flow rate was 0.4 mL min^{-1}, and the injection volume was 3 μL. The column temperature and the sample temperature were maintained at 40 °C and 5 °C, respectively.

For MS/MS analysis, the electrospray ionization source was operated in both positive (ESI^+) mode and negative (ESI^-) mode with the parameters set as follows: ion spray voltage, 5.5 kV (positive ion mode) and 4.5 kV (negative ion mode); desolvation temperature, 500 °C; source temperature, 150 °C. Nebulizing gas and desolvation gas flow rates were 7.0 bar and 1000 L/h, respectively. Multiple reaction monitoring (MRM) mode was developed for quantification of the targeted analytes. Data processing was performed using MultiQuant 3.0.2 (Analyst; AB SCIEX, Framingham, MA, USA). The parameters and the collision energies of precursor and product ions are listed in Supplementary Table S2.

Table S1. The demographic characteristics of participants in the three sampling areas.

	**All**	**Jiangsu**	**Zhejiang**	**Shanghai**
Total	227	76	76	75
Male	114	30	43	41
Female	113	46	33	34
Age (years)	45.4 ± 17.5	46.5 ± 15.1	51.9 ± 20.3	37.8 ± 13.4
BMI[1] (kg m^{-2})	23.1 ± 3.2	23.2 ± 3.1	23.1 ± 2.8	22.9 ± 3.6
Creatinine (mg dL^{-1})	131.5 ± 64.7	132.6 ± 59.5	127.7 ± 69.2	134.3 ±64.7

[1]BMI: body mass index.

Table S2. Co-occurrence of investigated mycotoxins and their metabolites in urine samples from Jiangsu, Zhejiang, and Shanghai.

**Table 1.**	**Distribution across location**			
	**Total Number (prevalence)**	**Jiangsu Number (prevalence)**	**Zhejiang Number (prevalence)**	**Shanghai Number (prevalence)**
2 Toxins	30 (13.22%)	11 (14.47%)	6 (7.89%)	13 (17.33%)
AFM1+DON-15-GlcA	2 (0.88%)	-[2]	-	2 (2.67%)
OTA+DON-15-GlcA	1 (0.44%)	-	-	-
OTA+ZEN	1 (0.44%)	-	1 (1.32%)	-
FB1+ZEN	2 (0.88%)	2 (2.63%)	-	-
FB1+DON-15-GlcA	1 (0.44%)	-	1 (1.32%)	-
DON+DON-15-GlcA	12 (5.29%)	2 (2.63%)	2 (2.63%)	8 (10.67%)
DON-3-GlcA+DON-15-GlcA	7 (3.08%)	3 (3.95%)	2 (2.63%)	2 (2.67%)
DON-15-GlcA+ZEN	4 (1.76%)	4 (5.26%)	-	-
3 Toxins	13 (5.73%)	5 (6.58%)	3 (3.95%)	5 (6.67%)
OTA+FB1+DON-15-GlcA	1 (0.44%)	-	-	1 (1.33%)
OTA+FB1+ZEN-14-GlcA	1 (0.44%)	-	-	1 (1.33%)
FB1+DON+DON-15-GlcA	1 (0.44%)	1 (1.32%)	-	-
DON-3-GlcA+DON-15-GlcA+ZEN	2 (0.88%)	2 (2.63%)	-	-
DON+DON-3-GlcA+DON-15-GlcA	5 (2.20%)	1 (1.32%)	1 (1.32%)	3 (4.00%)
DON-3-GlcA+DON-15-GlcA+ZEN-14-GlcA	1 (0.44%)	-	1 (1.32%)	-
DON+DON-15-GlcA+ZEN	2 (0.88%)	1 (1.32%)	1 (1.32%)	-
4 Toxins	8 (3.52%)	5 (6.58%)	2 (2.63%)	1 (1.33%)
AFM1+DON-3-GlcA+DON-15-GlcA+ZEN	1 (0.44%)	1 (1.32%)	-	-
AFM1+FB1+DON-3-GlcA+DON-15-GlcA	1 (0.44%)	-	1 (1.32%)	-
AFM1+FB1+DON+DON-15-GlcA	1 (0.44%)	-	-	1 (1.33%)
FB1+DON+DON-3-GlcA+DON-15-GlcA	2 (0.88%)	1 (1.32%)	1 (1.32%)	-
FB1+DON+DON-15-GlcA+ZEN	1 (0.44%)	1 (1.32%)	-	-
DON+DON-3-GlcA+DON-15-GlcA+ZEN	2 (0.88%)	2 (2.63%)	1 (1.32%)	-
Total	110 (48.46%)	43 (56.58%)	31 (40.79%)	36 (48.00%)

[1] Positive samples refer to urine samples containing mycotoxins with concentrations ≥ LOD.
[2] - , no sample.

Table S3. *P*-value of mycotoxin concentration distribution by age, sex, and BMI using the rank sum test (n = 227).

**Mycotoxins**	**Age[1]**	**Sex**	**BMI[2]**
AFM1	0.480	0.564	0.157
OTA	0.311	0.564	0.130
FB1	0.916	0.067	0.598
DON	0.712	0.162	0.375
DON-3-GlcA	0.221	0.370	0.678
DON-15-GlcA	0.508	0.137	0.506
ZEN	0.497	0.368	0.711
ZEN-14-GlcA	0.317	-[3]	0.317

[1]Including three age groups (20–45 years, 46–64 years and 65–88 years);
[2]Including three BMI categories (< 18.5, 18.5–23.9 and > 24 kg m^{-2}).
[3]*P*-value was not calculated since only two male urine samples were contaminated by ZEN-14-GlcA.

Table S4. Toxicities of FB1, OTA, DON, and ZEN from EFSA data.

Mycotoxin	Species	Duration	Critical effect	Dose (mg/kg bw/day)	TDI (μg/kg bw/day)	Reference
AFB1	Rat	Subchronic	Hepatocellular carcinomas	0.0004 ($BMDL_{10}$)	-	[1]
FB1	Mice	Chronic	Hepatotoxicity (increased incidence of megalocytic hepatocytes)	0.1 ($BMDL_{10}$)	1.0	[2]
OTA	Rat	Chronic	Neoplastic effect (kidney tumors)	0.0145 ($BMDL_{10}$)	-	[3]
DON	Mice	Chronic	Reduced body weight gain	0.11 ($BMDL_{05}$)	1.0	[4]
	Rat	Subacute	Estrogenic activity (increase in germ cell degeneration, sperm retention and abnormal nuclear morphology; effects on serum follicle-stimulating hormone (FSH) and luteinizing hormone (LH))	1 (NOAEL)	-	
ZEN	Pig	Chronic	Estrogenic activity (disturbance of the estrous cycle, ovulation, conception and implantation)	0.01 (NOEL)	0.25	[5]

Abbreviation: NOEL: no observed effect level; $BMDL_{10}$: the 90th percentile benchmark dose lower confidence limit; NOAEL: no observed adverse effect level.

Table S5. Food consumption in the total population stratified by location.

Food	24 h consumption (g or mL) (Mean ± SD)				P^1
	All	Jiangsu	Zhejiang	Shanghai	
Wheat	93.6 ± 103.0	80.6 ± 93.8	93.4 ± 107.1	107.0 ± 107.3	0.119
Maize	8.7 ± 33.1	7.9 ± 18.4	14.1 ± 52.2	4.0 ± 13.7	0.335
Rice	190.4 ± 119.4	176.3 ± 106.5	247.0 ± 126.4	147.3 ± 102.3	0.000*
Vegetables and fruit	179.9 ± 133.8	136.5 ± 79.5	206.6 ± 135.3	196.7 ± 163.6	0.015*
Meat	107.2 ± 92.9	109.2 ± 95.5	103.3 ± 101.3	109.0 ± 81.9	0.499
Nuts and seeds	24.4 ± 31.9	19.7 ± 24.6	29.6 ± 34.6	24.0 ± 35.0	0.189
Milk and dairy produce	47.1 ± 89.8	35.2 ± 80.0	49.3 ± 102.2	57.0 ± 85.6	0.022*
Beverages, coffee and tea	155.6 ± 213.6	249.0 ± 241.5	81.2 ± 166.5	136.3± 192.8	0.000*

[1]The *p*-value was calculated using the Kruskal–Wallis test, $*p < 0.05$.

Table S6. LC-MS/MS parameters for the detection of targeted mycotoxins.

Target	Molecular ion	Precursor ion (*m*/*z*)	Quantification ion (*m*/*z*)	CE[1] (Q) (eV)	Qualitative ion (*m*/*z*)	CE (q) (eV)	LOQ[2] (ng mL^{-1})	LOD[3] (ng mL^{-1})
AFB1	$[M+H]^+$	313	241	50	269	43	0.2	0.05
AFB2	$[M+H]^+$	315	259	38	287	34	0.5	0.2
AFG1	$[M+H]^+$	329	243	35	311	38	0.2	0.05
AFG2	$[M+H]^+$	331	245	40	285	37	0.5	0.2
AFM1	$[M+H]^+$	329	273	31	259	30	0.2	0.05
AFM2	$[M+H]^+$	331	189	54	216	48	0.2	0.05
OTA	$[M+H]^+$	404	239	34	221	22	0.1	0.05
OTα	$[M+H]^+$	257	102	40	221	22	0.1	0.05
FB1	$[M+H]^+$	722	334	50	352	50	0.2	0.1
DON	$[M+H]^+$	297	203	28	175	28	1	0.5
DON-3-GlcA	$[M-H]^-$	471	175	40	265	36	1	0.5
3-ADON	$[M+H]^+$	339	231	12	137	15	5	2
15-ADON	$[M+ H]^+$	339	261	12	279	10	5	2
T-2	$[M+H]^+$	484	305	14	185	18	0.3	0.1
HT-2	$[M+H]^+$	442	263	10	215	10	1	0.5
FUS-X	$[M+H]^+$	355	229	15	247	13	10	4
ZEN	$[M-H]^-$	317	175	35	131	40	0.2	0.1
ZEN-14-GlcA	$[M-H]^-$	493	317	40	175	40	0.5	0.2
α-ZEL	$[M-H]^-$	319	174	38	130	45	0.5	0.2
β-ZEL	$[M-H]^-$	319	174	38	130	45	0.5	0.2
α-ZAL	$[M-H]^-$	321	277	31	161	40	0.5	0.2
β-ZAL	$[M-H]^-$	321	277	31	161	40	0.5	0.2

[1]Collision energies (CE) (eV) required for optimal signal strength.
[2]LOD (limit of detection).
[3] LOQ (limit of quantification).

References

1. European Food Safety Authority (EFSA). Risk assessment of aflatoxins in food. *EFSA J.* **2020** *18*(3), 6040. https://doi.org/10.2903/j.efsa.2020.6040.
2. European Food Safety Authority (EFSA). Appropriateness to set a group health-based guidance value for fumonisins and their modified forms. *EFSA J.* **2018**, *16*(2):5172. https://doi.org/10.2903/j.efsa.2018.5172.
3. European Food Safety Authority (EFSA). Risk assessment of ochratoxin A in food. *EFSA J.* **2020**, *18*(5), 6113. https://doi.org/10.2903/j.efsa.2020.6113.
4. European Food Safety Authority (EFSA). Risks to human and animal health related to the presence of deoxynivalenol and its acetylated and modified forms in food and feed. *EFSA J.* **2017**, *15*(9):4718. https://doi.org/10.2903/j.efsa.2017.4718.
5. European Food Safety Authority (EFSA). Scientific opinion on the risks for public health related to the presence of zearalenone in food. *EFSA J.* **2011**, *9*(6), 2197. https://doi.org/10.2903/j.efsa.2011.2197.
